# Self-calibrating through-time spiral GRAPPA for real-time CMR

**DOI:** 10.1186/1532-429X-15-S1-E28

**Published:** 2013-01-30

**Authors:** Nicole Seiberlich, Mark Griswold

**Affiliations:** 1 Biomedical Engineering, Case Western Reserve University, Cleveland, OH, USA; 2 Radiology, Case Western Reserve University and University Hospitals of Cleveland, Cleveland, OH, USA

## Background

Through-Time non-Cartesian GRAPPA, a novel parallel imaging method for non-Cartesian trajectories, has recently been shown to provide real-time, free-breathing cardiac images with temporal resolutions of less than 35 ms per frame [Seiberlich N, *et al.* MRM 2011 Dec;66(6):1682-8]. The drawback to this method is the need for several fully-sampled datasets for calibration stemming from the non-Cartesian nature of the data, which leads to a longer overall scan time. By acquiring interleaved spiral datasets and combining them to form fully-sampled datasets for the generation of the GRAPPA weights, as in TGRAPPA [Breuer FA, *et al.* MRM 2005 Apr;53(4):981-5.], there is no need for additional calibration data. However, this interleaved calibration method poses the risk of increased artifacts if the temporal footprint of the calibration data is too long. The goal of this study is to test the extent to which self-calibrating through-time spiral GRAPPA can be used for real-time free-breathing CMR.

## Methods

Interleaved spiral data were acquired from healthy volunteers on a 1.5T Espree (Siemens Medical Solutions) using 18 receiver channels and the following parameters: bSSFP spiral sequence, TR = 4.48 ms, TE = 2.24 ms, FoV = 300 mm^2^, slice thickness = 8 mm, base matrix=128, 940 read-out points, 48 spiral arms made up of 6 sets of 8 arms, R=6, imaging time =17s. The temporal resolution for this scan was 35 ms/frame. The volunteers were instructed to breathe normally and no EKG gating was employed. The interleaved spiral data were resorted for use as calibration data; special care was taken to assure that the interleaf to be reconstructed was centered temporally within the calibration data to assure a short temporal window for calibration. Additionally, a separate calibration scan was acquired (48 arms, 80 frames) and the interleaved spiral data were also reconstructed using this data as a comparison. Finally, images were generated from the interleaved data using a sliding window view-sharing method for comparison.

## Results

**Figure 1:**
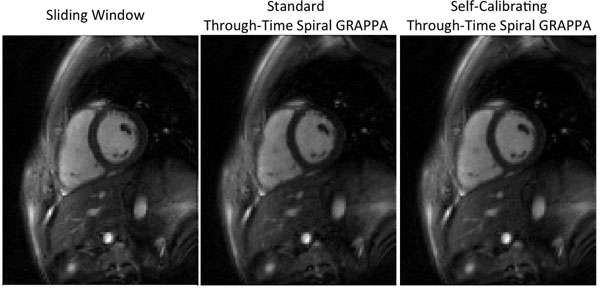
Representative images of the heart in diastole reconstructed from interleaved spiral data using (left) sliding window, (center) standard through-time spiral GRAPPA, and (right) self-calibrating through-time spiral GRAPPA.

**Figure 2:**
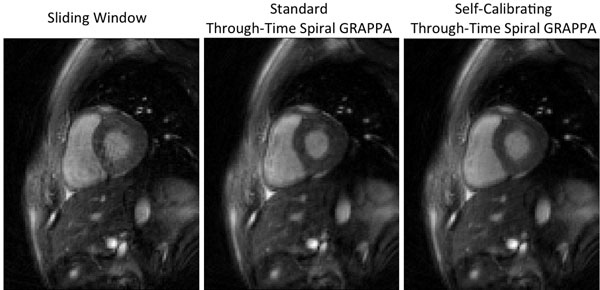
Representative images of the heart in systole reconstructed from interleaved spiral data using (left) sliding window, (center) standard through-time spiral GRAPPA, and (right) self-calibrating through-time spiral GRAPPA.

The results of the different reconstruction methods for both diastole and systole are shown in Figures 1 and 2; sliding window images are shown on the left, standard through-time spiral GRAPPA images generated using a calibration dataset are center, and self-calibrating through-time spiral GRAPPA images are on the right. During diastole, where there is not significant cardiac motion, all three reconstruction techniques performed well. The through-time spiral GRAPPA images demonstrate nearly identical image quality, and clearly fewer motion artifacts as compared to the sliding window reconstruction. While a separate calibration dataset was required to generate the center image of Figure 2, no additional data or time were required to arrive at the artifact-free image at the right of Figure 2.

## Conclusions

Self-Calibrating Through-Time Spiral GRAPPA can be used to generate real-time cardiac images with a temporal resolution of 35 ms without the need for an additional calibration dataset.

## Funding

This work was funded by Case Western Reserve University/Cleveland Clinic CTSA UL1 RR024989, NIH/NIBIB R00EB011527 and RO1HL094557.

